# Bovine serum albumin under the influence of alkali metal halides

**DOI:** 10.1039/d4ra04503c

**Published:** 2025-01-02

**Authors:** Yojana J. P. Carreón, A. M. Jaramillo-Granada, D. Fuentes-López, A. D. Reyes-Figueroa, J. González-Gutiérrez, H. Mercado-Uribe

**Affiliations:** a Facultad de Ciencias en Física y Matemáticas, Universidad Autónoma de Chiapas Tuxtla Gutiérrez Chiapas 29050 Mexico; b CONAHCYT CDMX Mexico; c CINVESTAV-Monterrey, PIIT Apodaca Nuevo León 66628 Mexico hmercado@cinvestav.mx; d CIMAT-Monterrey, PIIT Apodaca Nuevo León 66628 Mexico

## Abstract

The hydration shell of a protein is so important and an integral part of it, that protein's structure, stability and functionality cannot be conceived in its absence. This layer has unique properties not found in bulk water. However, ions, always present in the protein environment, disturb the hydration shell depending on their nature and concentration. In this work, we study the effect of four alkali metal halides (LiCl, NaCl, KCl and CsCl) on a Bovine Serum Albumin (BSA) suspension. In order to investigate the influence of such ions on this protein, we use several experimental methods: dynamic light scattering, differential scanning calorimetry, thermogravimetry, Fourier transform infrared spectroscopy and image analysis. We found that Li^+^ and Na^+^ prevent protein aggregation. Moreover, the ion size affects the interaction with the secondary structure of the protein (Amide III band). Notably, for the smallest ion (Li^+^), the water–ion interaction dominates over the Amide A band signature, contrasting with the other ions. We also differentiate between bulk and hydration water through the evaporation of protein suspensions.

## Abstract 2

Particle size distribution of BSA and BSA with different salts.

## Graphical abstract

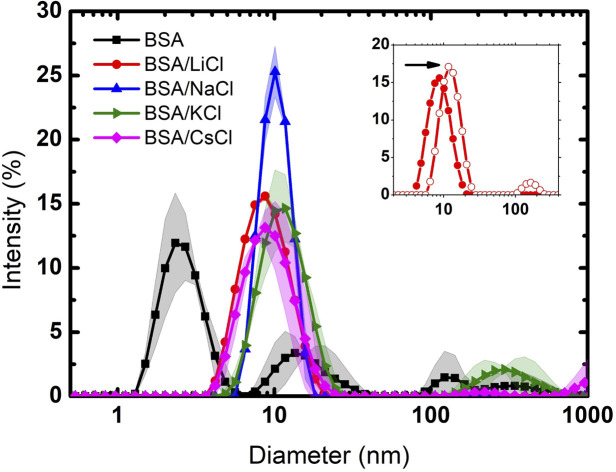

## 1 Introduction

Ions play a crucial role in biological processes, participating in signaling, homeostasis, metabolic, and chemical reactions.^1–3^ For instance, the digestive function and programmed death in cells are carried out by lysosomes, which work using enzymes in an environment with a given ionic balance;^1^ some mechanical and chemical stimuli are activated by potassium ions involved in sensory neurons.^2^ Additionally, ions are critical for crosslinking in cytoskeletal filaments and influencing protein solubility.^3^

Water molecules are equally important in biological processes, forming a dynamic network that supports various functions. Water molecules are oriented according to the superficial charge of proteins and form a hydration layer which is harmoniously driven by hydrogen bonds. The addition of salts in the protein medium produces modifications in such a layer. Depending on the nature and concentration of these salts, is the disturbance produced.^4,5^

There is a common classification of ions that is worth to remark here in order to understand why and how they affect the interaction of biomolecules. Ions are classified as kosmotropes or chaotropes. Kosmotropes are small ions with a high charge density and water molecules strongly bind to them. In contrast, chaotropes are big ions with low charge density so they weakly bind to water molecules.^5–7^ Initially, it was considered that their effects were indirectly related to ion's ability to “make” or “break” bulk water structure. However, current models are based on a direct dependence of the interactions between ions and the first hydration layer of a macromolecule. For instance, in the presence of a chaotrope agent like urea, water molecules compete for the internal hydrogen bonds and the hydration shell of a protein, resulting in destabilization and unfolding of the protein. This event brings with it an increase in the degrees of freedom of water molecules, and consequently, an increase in entropy.^8,9^

Kosmotropes are proposed to enhance hydration shells, thereby increasing protein stability while reducing solubility; chaotropes weaken the hydration shells and decrease protein stability.^10^ In other words, kosmotropic ions compete for interfacial water on the protein surface, thus producing a poorer solvent, while chaotropic cations improve the solvent.^11^ Small organic solvents, also considered as cosolvents, have the ability to induce changes in the protein hydration layer.^5,12^

A very important issue is related to protein crystallization. For this process to occur, two crucial elements are needed: adding ions to produce a neutral complex and dehydrating the surface of the protein one wants to crystallize.^11^ Hence, kosmotrope ions help to produce crystallization.

Beyond the context of proteins, the dynamics of hydration–dehydration in the presence of ions are significant in various industries, such as pharmaceuticals and food processing. Modifications in material properties, such as swelling, wettability, solubility, foamability, gelation, emulsification, and degradation, are influenced by hydration and dehydration processes.^13–15^

Hydration involves adding water molecules to a dry polymer,^16^ continuing until no further changes occur. This addition can be carried out through the contact between a polymer and water molecules in the liquid or gaseous phase; and it finishes once the water molecules do not produce further changes in the polymer.^16^ In contrast, dehydration consists of the loss of water molecules either in a slow or fast way.

In order to investigate the interactions between water molecules and proteins as well as the protein itself, infrared spectra of partially hydrated proteins have been investigated. The advantage of this method is the great sensitivity of the vibrational modes of hydrogen bonds.^16,17^ Upon drying droplets of biological fluids^18^ and biopolymer solutions,^19,20^ films form on the substrate's surface, with textures reflecting the solutes' properties and interactions. Variations in surface morphology reveal interactions with ions present in the initial solution, serving as a model for understanding similar interactions *in vivo*.^21^ The complex textures of these films often result from crystalline hydrates involving proteins and salts, formed due to capillary flows during the evaporation process, leading to distinctive morphological changes. Previous studies, particularly with solutions containing bovine serum albumin (BSA) and salts, have shown that these intricate structures are driven by electrostatic interactions within the water–biopolymer–ion system, forming supramolecular structures within specific concentration ranges.^21–23^

In this paper, we investigated how different alkali metal cations (Li^+^, Na^+^, K^+^, and Cs^+^) affect the hydration shell and overall structure of Bovine Serum Albumin (BSA) suspensions. Since BSA is an acidic protein, with pH 4.7, is highly soluble in water at pH around 7 (which is the condition in our experiments). Therefore, each one of the above cations must affect such negative protein.

With the aim to elucidate the specific interactions between different alkali metal ions and BSA, we used dynamical light scattering, differential scanning calorimetry, thermogravimetry, Fourier transform infrared spectroscopy and image analysis of dried droplets. We found that ion size affects the interaction with the secondary structure of the protein (Amide III band) and the calorimetric profiles. Moreover, depending on the ion the morphology and texture of the dry droplet patterns is different. Our findings could provide valuable insights for understanding and controlling hydration–dehydration dynamics of proteins.

## 2 Materials and methods

### 2.1 Protein preparation

High purity bovine serum albumin (BSA), lithium chloride (LiCl), sodium chloride (NaCl), potassium chloride (KCl) and cesium chloride (CsCl) (all of them supplied by Sigma-Aldrich) were used to prepare stock solutions. These compounds were dissolved in deionized water (Mili-Q, *R* = 18.2 MΩ cm) at pH 6.8 and 25 °C. The stock solutions were diluted according to the desired concentration: protein, *C*_p_ = 120 μM (8 mg mL^−1^) and salt, *C* = 75 mM. The solutions were stored at 2 °C and, before use, they were thermalized at room temperature.

### 2.2 DLS measurements

Dynamic light scattering (DLS) measurements were carried out using a Malvern Zetasizer Nano ZSP. The protein suspension (1 mL) was poured into a disposable polystyrene cuvette and exposed to a 633 nm laser at 25 °C. A stabilization time of 120 s was given. Three independent measurements were performed, each one with twelve iterations, and the average was obtained.

### 2.3 DSC measurements

Differential scanning calorimetry measurements were performed using a microcalorimeter NanoDSC (TA, Instruments). The salt-protein sample was compared with a control sample (BSA) suspended in deionized water. The samples were loaded into the capillaries of the equipment to obtain heat capacity profiles at 3 atm, equilibrium time of 5 min and a scan rate of 1 °C min^−1^. The analysis of the calorimetric spectra was done with the software provided by the instrument. Due to the high reproducibility, the experiments were performed twice using different samples.

### 2.4 Thermogravimetry measurements

The measurements were carried out using a Q500 Thermogravimetric Analyzer (TA, Instruments) with a nitrogen flux (60 mL min^−1^). A drop (16 μL, approximately) for each case was poured on a platinum pan to analyze its evaporation process by isothermal assays.^24^ Every sample was measured at 50 °C until its total evaporation. We also evaluated the weight loss of pure water under the same conditions as a reference. The weight loss and first derivatives of the samples were recorded for each case. At least, two independent measurements were performed. The time axis was shifted to set the initial mass at 12 mg.

### 2.5 FTIR measurements

Fourier transform infrared (FTIR) spectra were recorded on a Jasco FT/IR-4700 spectrometer. Protein films were formed on ZnSe windows. A 10 μL drop of the solution was deposited on a clean ZnSe window, which is transparent for wave numbers higher than 600 cm^−1^. This material is not hygroscopic, is not water soluble and it can be used with samples that contain liquid water. The drop deposited is allowed to evaporate under controlled environmental conditions: *T* = 25 °C and relative humidity of 55%. Then, the samples were rehydrated and placed on a holder for their measurements. For each spectrum, an interferogram of 66 scans with a resolution of 4 cm^−1^ was measured in the region 4000 to 600 cm^−1^. This process was repeated five times for each sample. This number of experiments allows us to ensure reproducibility. It is important to note that the stain left by the drop is fully illuminated by the infrared laser. In a separate experiment, the spectrum of the window without sample was measured as the reference signal under identical conditions.

### 2.6 Spectra analysis

The FTIR spectra were analyzed in the program OriginLab, version 2016. A baseline correction was made for each spectrum and the reference was subtracted. The resulting differentiated spectra were analyzed with a three order polynomial and softened using a seven-point Savitsky–Golay function^25^ to eliminate possible white noise. This protocol allows us to obtain well-defined spectra of protein suspensions according to their hydration level.

### 2.7 Image analysis

The structural analysis of the droplets patterns were performed by the radial density profile *I*(*r*). This parameter describes a profile of integrated intensities generated by concentric circles as a function of the radial distance and it is given in 2D by the equation:^26^
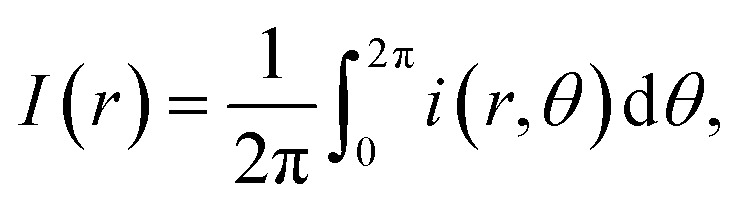
where *i*(*r*) is the local light intensity inside a circle of radius *r*, and *I*(*r*) is the sum of the pixel intensities around the circle. The image analysis shows a high reliability because produces similar results for each sample.

## 3 Results and discussion

### 3.1 Particle size distribution

**Fig. 1:**
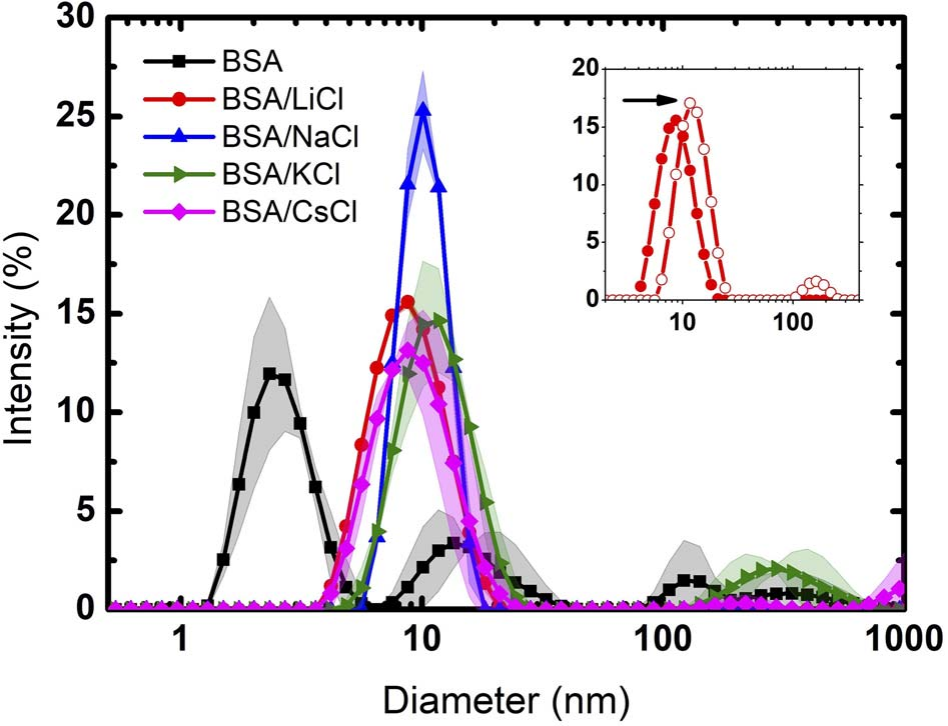
Particle size distribution of BSA and BSA with different salts. The solid lines are the averages, and the bands the dispersions of the data from the experiments.

Fig. 1 depicts the diameter of BSA and BSA/salt in the presence of different chloride salts (LiCl, NaCl, KCl, CsCl). Even though at the pH used (≈7) the BSA protein is presumably dissolved as a monomer, its polydisperse nature is evident. Such polydispersity may be explained by the high charge distribution which leads to aggregation (impossible to remove even using high power sonication). In this context, Singh *et al.*^27^ reported the protein size in solutions at pH 4.5 and pH 7.0. Their DLS measurements showed protein aggregation at pH 4.5 with size distributions centered at 5, 10 20 and 50 nm. At pH 7.0, the sizes centered only around 5 and 10 nm. In our measurements, the small size distribution centered at 3 nm is due perhaps to dissolved peptides or impurity tracers. Note that such peptide fragments are only seen when the medium is Milli Q water. Once the salts are added (75 mM), the size distribution of these fragments disappears and monomers between 7 and 10 nm show up. The reason is due to Rayleigh dispersion, where the intensity of the light scattered in DLS experiments goes as *d*^6^ (*d* is the diameter of the scatterers).^28^ Indeed, the intensity produced by monomers increases between 160 and 1300 times, making the contribution of the small peptides insignificant.

Clearly, only Li and Na were able to produce pure monomer distributions, indicating a full salting-in behaviour. Since both ions are considered to be kosmotropic agents, this result is contrary to what would be expected for hydrophobic molecules (where such ions give rise to salting-out effects). In the case of this highly charged protein, the cations screen only the negatively charged patches and the result is full solvation due to repulsive electrostatic forces by positive residues.

At the given concentration (75 mM), the low density charge of the chloride ions is not enough to neutralize the positive charges of the protein. However, at 300 mM (4 times larger than the previous one), chloride ions are now able to neutralize the positive charge. Protein aggregation, due to entropic and van der Waals forces, takes place as seen in the inset of Fig. 1 for the case of LiCl. The diameter of the size distribution doubles, corresponding to dimer molecules.

K and Cs cations, which are considered to be chaotropic, do not fully disaggregate BSA proteins. Again, while chaotropic ions give rise to salting-in effects, in the case of BSA such solvation is not fully observed. In such situation, big cations (depending on concentration) are located preferably at the solute hydrated surface. On the other hand, small cations, which are highly hydrated, can permeate such layer.^29^

### 3.2 Calorimetric profiles

**Fig. 2:**
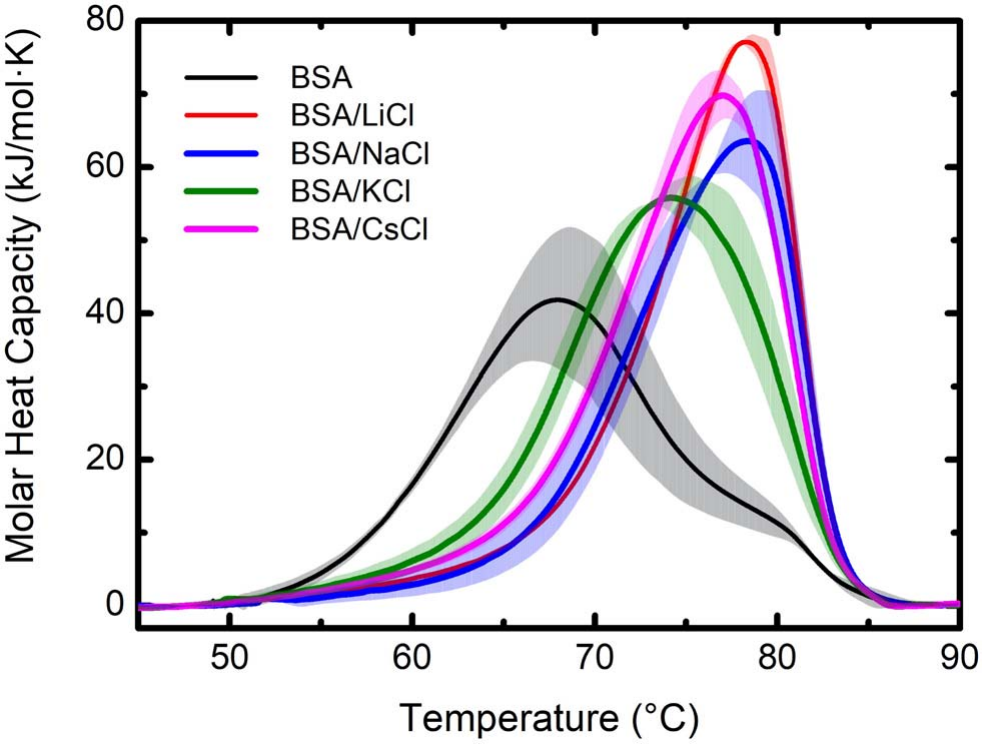
Calorimetric profiles of BSA and BSA/salt mixtures at a concentration of *C*_p_ = 120 μM (8 mg mL^−1^) and *C* = 75 mM, respectively. The solid lines are the averages, and the bands the dispersions of the data from the experiments.

DSC analysis provides insights into the thermal stability and unfolding behavior of BSA in various ionic environments. The DSC thermogram of BSA without any added salt shows a distinctive peak centered around 68 °C, see Fig. 2. This peak represents the thermal denaturation temperature of BSA, indicating its transition from the folded to the unfolded state. It is important to remark that the thermogram is very wide, indicating that before unfolding there is first a disaggregation process. In contrast, upon the addition of the salts, the calorimetric profiles suffer a significant modification, not only in height but in form and thermal response. Note that for LiCl and NaCl, the profiles are narrower because the suspensions are fully monodisperse. The corresponding enthalpies are: BSA (642); LiCl (783); NaCl (724); KCl (777) and CsCl (810), all in kJ mol^−1^.

Furthermore, recently, Lau and Bilodeau^30^ reported that Li^+^ forms tight clusters with a long-range ordering on the surface of capto-ligands used for chromatography. Contrarily, larger cations, with lower charge densities like Cs^+^, exhibit unbounded and less ordered interactions. BSA is a protein made of three domains and three hydrophobic cavities where different ligands can attach. Usually the bigger ions interact on the surface of the protein disturbing its hydration layer.^5,10^

### 3.3 Evaporation by thermogravimetry

**Fig. 3:**
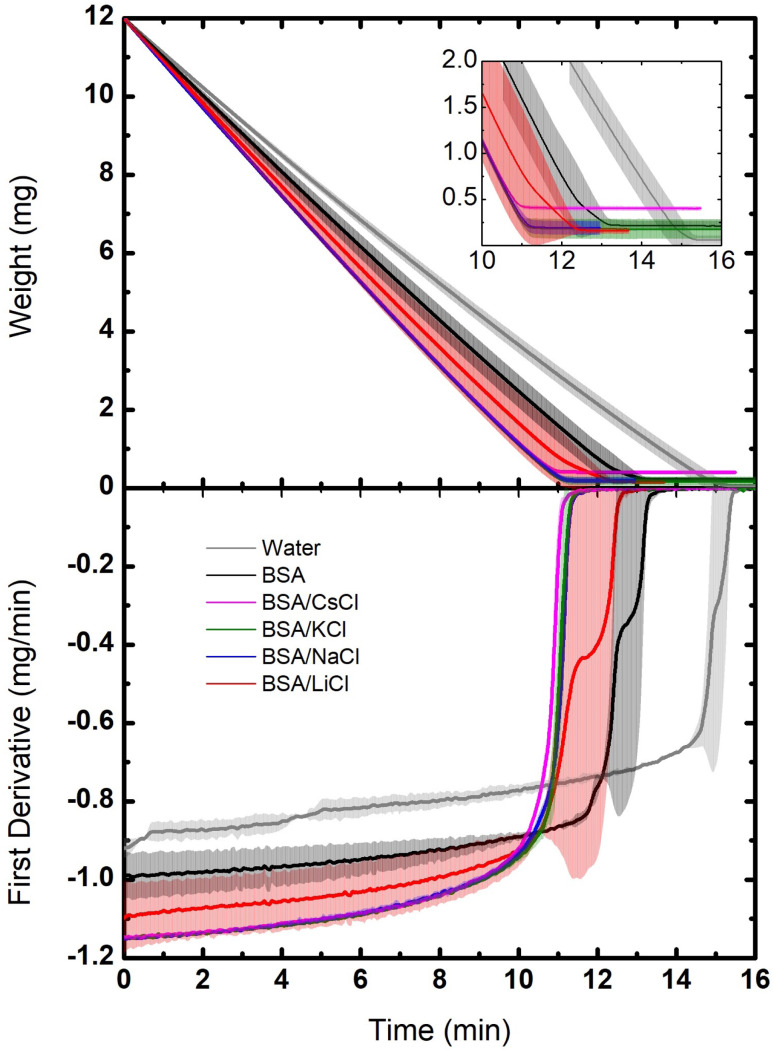
Dynamic of the dehydration for BSA suspensions with different salts: LiCl, NaCl, KCl, and CsCl at *C* = 75 mM and *C*_p_ = 120 μM (8 mg mL^−1^). The samples were measured at 50 °C with a constant helium flux (60 mL min^−1^). Top: weight loss as a function of time. Bottom: derivative of the data as a function of time. The evaporation of a water sample is plotted as reference (gray lines). The solid lines are the averages, and the bands the dispersions of the data from the experiments.

Evaporation of small drops of the protein suspensions were also studied by thermogravimetry in isothermal conditions. These experiments provide important information about dehydration, revealing differences in molecular interactions between water, ions and proteins. Fig. 3 shows our results, compared to a control sample using pure water (Milli-Q water, 18.2 MΩ cm). It can be observed that the rate of weight loss is constant in all cases, except at the end of the evaporation process. In other words, the dynamics of evaporation in the suspensions is dominated by the loss of free water. However, at the end of the process, when free water is gone, the rate of weight loss slows down (see inset). This is due to the fact that the last remaining water molecules interact strongly with proteins and ions, making it more difficult to go to air.^24^ The portions of the TGA curves corresponding to the evaporation of free water includes from the beginning of the evaporation (12 mg) to the point where the slope starts to change (0.2 mg). Considering that one mol of water is 18 g, free water corresponds then to 0.65 × 10^−3^ mol and bound water to 5.8 μmol.

In Fig. 3 (bottom) such dynamics is better observed through the derivative of the weight loss. The drop that takes longer to evaporate was the one of pure water, then the BSA suspension, followed by the drops of BSA/salts suspensions. Since in the latter cases the free water is minimal (the ions are highly hydrated and the hydration of the BSA is enhanced), they evaporate faster. Indeed, the suspension of BSA proteins with no salts keep only their own hydration layers, so the amount of free water is larger. Instead, the largest amount of free water molecules is found in the drop of pure water, which is why it takes the longest to evaporate.

### 3.4 FTIR spectra of films

**Fig. 4:**
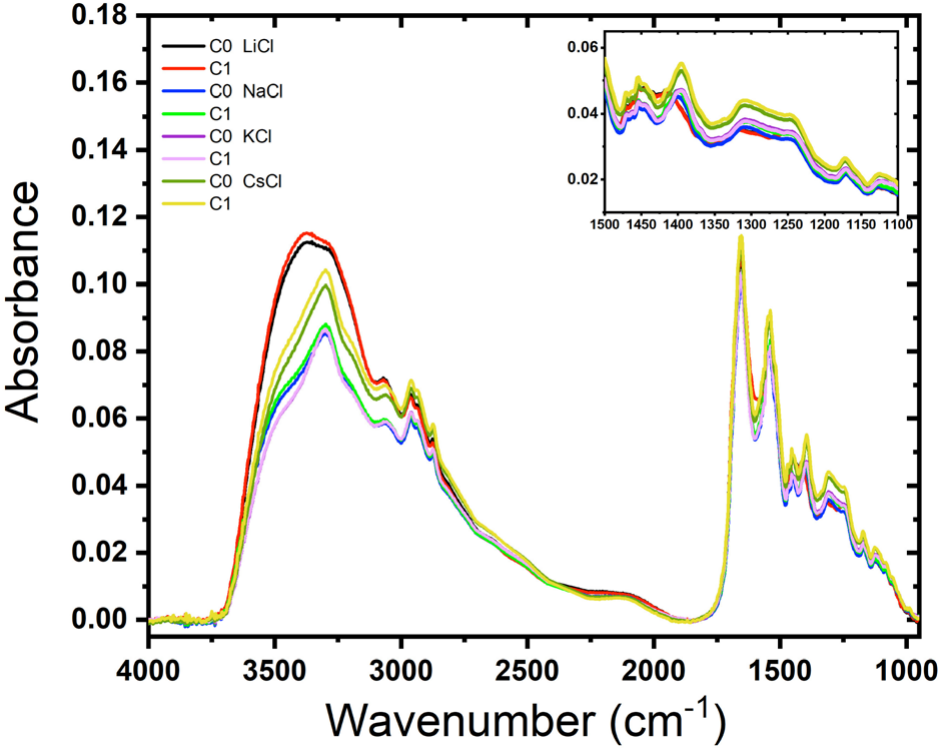
Effect of rehydration of BSA films containing different salts. Absorbance as a function of the wave number of BSA films (*C*_p_ = 120 μM (8 mg mL^−1^)) with salts: LiCl, NaCl, KCl and CsCl (*C* = 75 mM).

We examined the role of the different ions in the hydration and rehydration process of the BSA suspensions. Fig. 4 shows the representative infrared spectra of almost dried droplets from solutions of BSA with the salts after their deposition on a ZnSe window. The labels C0 represents the initial dry drop (cycle 0), and C1 represents the same drop after rehydration and subsequent evaporation (cycle 1). As depicted in the figure, the shape of the spectra are similar for almost all the films but it is clear that the order does not follow the sequence of Hofmeister series, except in the complex band Amide III (around 1300 cm^−1^) that is highly specific for distinct molecules (see the inset in the image). It is interesting to note that LiCl produces the widest peak (O–H bond stretching), which is distinctive of water, indicating that Li^+^ ions significantly alter the hydrogen bonding network. Since Li^+^ is the most kosmotropic ion, its interaction with water is so predominant than the protein signal is fairly obscured.

The spectra for BSA with NaCl show similar shapes in the O–H stretching region to those of KCl and CsCl, but with different absorbance values. This indicates that Na^+^ ions affect the hydration and hydrogen bonding differently. The spectra for BSA with KCl show minimal differences between C0 and C1, indicating that the rehydration and subsequent evaporation process does not significantly alter the protein's hydration. This suggests that K^+^ ions provide stabilization to the protein structure, consistent with findings that K^+^ does not facilitate hydrogen bond formation.^30^ On the other hand, rehydration affects only CsCl, reflecting its chaotropic nature. The spectral changes upon rehydration and evaporation indicate notable conformational alterations due to the narrow layer of water molecules surrounding Cs^+^ ions. Therefore, just a small amount of water added to the dried droplet is enough to see some difference resulting in a narrow signal in the region between 3250–3750 cm^−1^. On the other hand, the Amide III band does not depend on the rehydration event because this band it is not affected by water absorption.^31^ The peak at 1650 cm^−1^ corresponds to the Amide I that overlaps with the scissor bend of water. These results agree with those obtained by Bridelli,^32^ where water deprivation in proteins modifies the lateral group. In the study of hydration in globular proteins, Buontempo and collaborators were pioneers in the use of infrared spectroscopy.^33^ They analyzed the spectra of protein films before and after a dehydration process. Their study showed the presence of water molecules that interact with each other and the surface of the protein in the region 3300–3000 cm^−1^. Then, it was possible to distinguish a contribution due to liquid and tightly bound water. More recently, Bridelli studied the hydration in proteins films, globular (collagen) and fibrous (lysozyme), changing the relative humidity conditions. The conclusion of the work was that water deprivation in proteins induces structural rearrangements and modifications in the exposure of the lateral chain groups.

### 3.5 Dried droplet patterns of BSA suspensions

The thermogravimetry experiments gave us information about the evaporation dynamics of the suspension droplets, where it is clear that bulk (free) water evaporates differently than bound water depending on the salt. Now, we would like to evaluate the final patterns left by such evaporation events.

Drying a droplet of protein solution generates a uniform deposit surrounded by a well-known ring-shaped structure,^34,35^ called the coffee ring effect. It is produced by capillary flows that emerge from the evaporation of water molecules in the contact line of the droplet.^36–38^

**Fig. 5:**
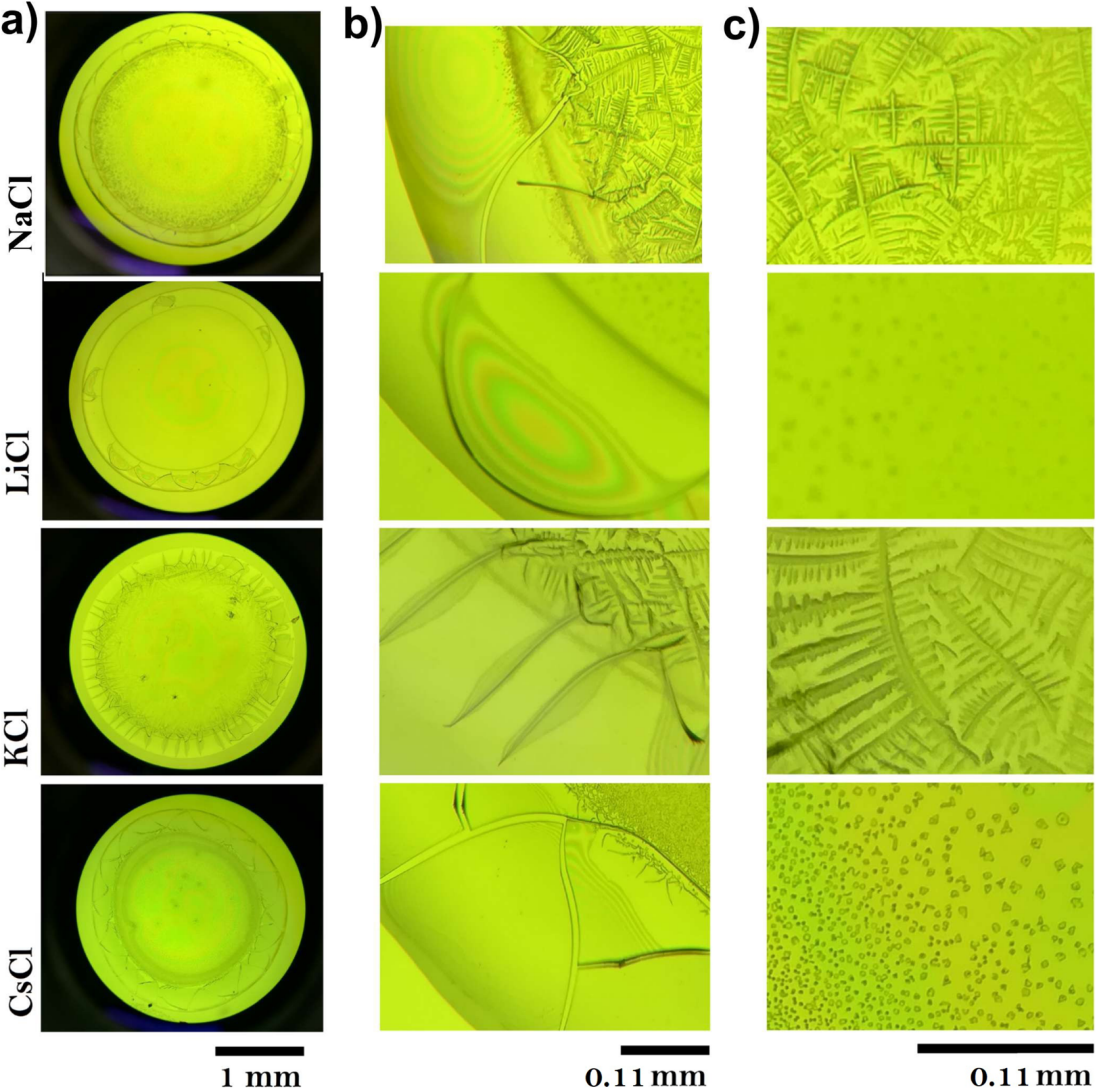
Dried droplets of BSA suspensions with different salts. (a) Deposit patterns produced by NaCl, LiCl, KCl, and CsCl (*C* = 75 mM and *C*_p_ = 120 μM). (b) The coffee ring of the deposits patterns. (c) A zoom of the central region in the deposits.

Fig. 5 displays dried droplets of the above BSA/salt suspensions. The deposits exhibit clear differences in their structure. Panel (a) shows the overall views of the dried droplets, each exhibiting distinctive patterns based on the salt used. Panel (b) focuses on the border regions of the droplets. The droplet with LiCl displays a uniform distribution with a faint coffee-ring pattern, indicating complete solvation of the BSA protein, as observed in DLS experiments. The NaCl droplet shows a pronounced coffee ring effect with significant material accumulation at the edges, suggesting a migration of protein and salt towards the periphery during drying.

Regardless the ion, cracks appear in the coffee rings, although they are more pronounced for KCl, as shown in Fig. 5b. Panel (c) highlights the central parts of the droplets. The LiCl central region shows a relatively homogeneous distribution of fine particles, suggesting material transport to the edges during drying. The central part of the NaCl droplet is marked by a homogeneous distribution of crystalline structures, forming complex branched structures. These structures suggest strong localized interactions between NaCl and BSA, leading to effective nucleation and growth processes. The morphology indicates that NaCl contributes to a balanced evaporation process, maintaining a uniform distribution of material across the droplet. The KCl droplet's center is densely packed with complex crystalline formations. This high degree of crystallinity indicates strong ionic interactions and effective nucleation processes within the droplet. The central part of the CsCl droplet displays a sparse and uniform distribution of particles. The lack of pronounced crystalline structures points to a more even evaporation process, likely influenced by the weaker ionic interactions of CsCl with BSA. The distinct patterns observed in the dried BSA droplets are a direct consequence of the interactions between the chloride salts and the protein. On the other hand, LiCl produces uniform coatings inside the deposits, while NaCl and KCl induce the formation of complex aggregates, and CsCl generates small amorphous aggregates (see panel c). The structural changes in deposits of proteins during its evaporation have been studied by different groups.^39–41^ It has been reported that mass transport mechanisms are affected by Marangoni flows, which avoid the formation of the coffee ring.^42–45^ They are produced by surfactant and temperature gradients that induce inward recirculation in the droplet. The final morphology of patterns depends on the aggregation processes which are driven by forces such as friction Fdrag, electrostatic force Fex (caused by the charges of the molecules), and adhesion Fad (between the macromolecules and the glass substrate).^38,46^ For instance, cracks in deposits evolve to complex patterns by mixing protein and liquid crystals.^40,41^ Mixtures of two proteins produce fractal-like structures in the interior of the deposits.^39^ Other authors have also showed that the addition of salts in protein suspensions induces the formation of complex patterns such as amorphous peripheral ring, dendritic shapes, zigzag patterns, among many others.^20,47–55^

### 3.6 Radial density profiles

**Fig. 6:**
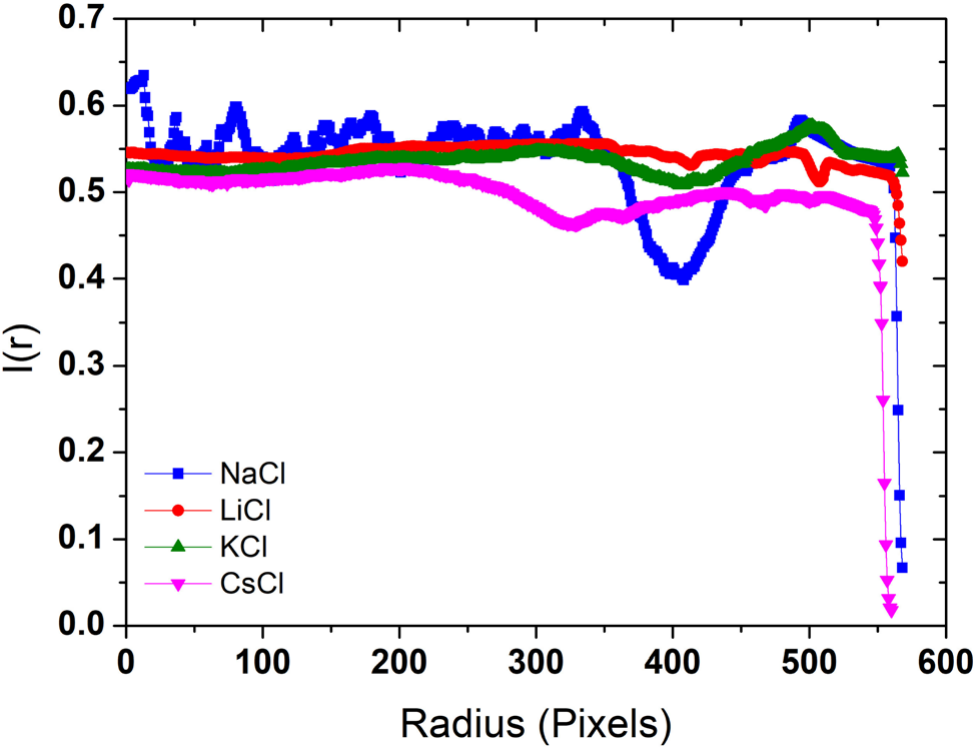
Radial density profiles of the deposits formed by dried droplets in Fig. 5.

The radial density profile is a way to capture the distribution of BSA within the dried droplets influenced by the salts. In Fig. 6 we show the radial density profile for the corresponding deposits depicted in Fig. 5. The profile for LiCl is relatively flat across the radius, indicating a more uniform distribution of material. The lack of significant peaks is consistent with the smooth patterns observed in the droplet images, suggesting even drying dynamics and balanced interactions between BSA and Li^+^ ions. In contrast, the radial density profile for NaCl exhibits a pronounced peak near the edge of the droplet, indicating a strong coffee ring effect. This suggests significant peripheral accumulation of material, aligning with the visual observations of pronounced coffee-ring formations in the dried droplet images, and reflecting the migration of BSA towards the periphery during the drying process. The profile for KCl shows moderate variations with a slight increase towards the edge but no distinct peak, indicating a combination of central and peripheral deposition that reflects the mixed patterns of coffee ring and crystalline structures observed in the droplet images. This highlights the complex interaction between K^+^ ions and BSA during the drying process. The profile for CsCl is relatively flat with some fluctuations, but with an overall decrease in intensity towards the edge, suggesting disrupted and less organized deposition. This is consistent with the visual observation of irregular patterns and scattered deposits. The chaotropic nature of Cs^+^ ions likely leads to less structured and more scattered deposits, indicating that Cs^+^ ions disrupt the organized deposition of BSA. These radial density profiles highlight the distinct effects of each chloride salt on the distribution and deposition patterns of BSA during the drying process.

## 4 Conclusions

The hydration layer of proteins is crucial to maintain their structure, stability and proper functionality. The presence of ions in a protein solution has different effects depending on their nature and concentration. In order to investigate the effect on BSA proteins, we have used alkali metal salts with different charge densities. We employed various methods to obtain complementary information about this effect. We found that full salting-in of BSA protein suspensions takes place at 75 mM of LiCl and NaCl. However, at a four-fold higher concentration of chloride ions (300 mM) the positive residues are neutralized and dimers are formed. Our DSC findings clearly indicate the change in electrostatic interaction of the aggregated state of BSA and the solubilization of BSA in the presence of salts (more notoriously for the case of the kosmotropic ions).

Moreover, the ion size affects the interaction with the secondary structure of the protein (Amide III band). In the case of the smallest one (Li^+^) the interactions between water and ion are more significant than the fingerprint of Amide A. We learnt that LiCl is the salt that more affects the water intake of the protein; not only gives rise to a less complex pattern after dehydration but to a smaller protein absorption. Our findings provide valuable information for understanding the interactions involved during protein hydration in the presence of different types of cationic ions.

## Data availability

We declare that the data obtained in this work were generated during the reported experiments in the manuscript and all of them are included in it.

## Author contributions

Y. J. P. C.: conceptualization, experiments, data analysis, software, investigation, visualization, writing—review and editing. A. M. J. G.: experiments and data analysis. D. F. L.: experiments. A. D. R. F.: software and data analysis. J. G. G.: software, data analysis, visualization and writing—original. H. M. U.: conceptualization, experiments, data analysis, formal analysis, funding acquisition, supervision, writing original, and writing—review and editing.

## Conflicts of interest

There are no conflicts to declare.

## References

[cit1] Trivedi P. C., Bartlett J. J., Pulinilkunnil T. (2020). Cells.

[cit2] Kefauver J., Ward A., Patapoutian A. (2020). Nature.

[cit3] Kozawa S. K., Wnek G. E. (2021). Polym. Int..

[cit4] Arakawa T., Timasheff S. N. (1984). Biochemistry.

[cit5] Ball P., Hallsworth J. E. (2015). Phys. Chem. Chem. Phys..

[cit6] Zangi R. (2010). J. Phys. Chem. B.

[cit7] Zhang Y., Cremer P. S. (2006). Curr. Opin. Chem. Biol..

[cit8] Schiffer C. A., Dötsch V. (1996). Curr. Opin. Biotechnol..

[cit9] Timson D. J. (2020). World J. Microbiol. Biotechnol..

[cit10] Casanova-Morales N., Alavi Z., Wilson C. A., Zocchi G. (2018). J. Phys. Chem. B.

[cit11] Collins K. D. (2019). Q. Rev. Biophys..

[cit12] Canchi D. R., García A. E. (2013). Annu. Rev. Phys. Chem..

[cit13] Lawrence B. D., Wharram S., Kluge J. A., Leisk G. G., Omenetto F. G., Rosenblatt M. I., Kaplan D. L. (2010). Macromol. Biosci..

[cit14] O'Neill H., Pingali S., Petridis L., He J., Mamontov E., Hing L., Urba V., Evans B., Langan P., Smith J. (2017). *et al.*, Dynamics of water bound to crystalline cellulose. Sci. Rep..

[cit15] Asghar A., Henrickson R. (1982). Adv. Food Res..

[cit16] Rupley J. A., Careri G. (1991). Adv. Protein Chem..

[cit17] Careri G., Giansanti A., Gratton E., Snamprogetti (1979). Biopolymers.

[cit18] Arévalo L. A., O'Brien S. A., Antonova O., Seifert A. (2022). J. Phys.: Conf. Ser..

[cit19] Carreón Y. J., Gómez-López M. L., Díaz-Hernández O., Vazquez-Vergara P., Moctezuma R. E., Saniger J. M., González-Gutiérrez J. (2022). Sensors.

[cit20] Glibitskiy G., Glibitskiy D., Gorobchenko O., Nikolov O., Roshal A., Semenov M., Gasan A. (2015). Nanoscale Res. Lett..

[cit21] Glibitskiy D., Gorobchenko O., Nikolov O., Cheipesh T., Dzhimieva T., Zaitseva I., Roshal A., Semenov M., Glibitskiy G. (2023). Sci. Rep..

[cit22] Gorobchenko O., Glibitskiy D., Nikolov O., Cheipesh T., Dzhimieva T., Zaitseva I., Roshal A., Semenov M., Glibitskiy G. (2024). Low Temp. Phys..

[cit23] Biglari G., Saberi M., Issakhani S., Jadidi O., Farhadi J., Bazargan V., Marengo M. (2023). Macromol. Mater. Eng..

[cit24] Hernández-Galván G., Mercado-Uribe H. (2023). Soft Matter.

[cit25] Savitzky A., Golay M. J. (1964). Anal. Chem..

[cit26] González-Gutiérrez J., Pérez-Isidoro R., Pérez-Camacho M., Ruiz-Suárez J. (2017). Colloids Surf., B.

[cit27] Raghuwanshi V. S., Yu B., Browne C., Garnier G. (2020). Front. Bioeng. Biotechnol..

[cit28] Velez-Saboyá C., Guzmán-Sepúlveda J., Ruiz-Suárez J. (2022). J. Phys.: Condens. Matter.

[cit29] Parsons D. F., Boström M., Nostro P. L., Ninham B. W. (2011). Phys. Chem. Chem. Phys..

[cit30] Lau S. C., Bilodeau C. L. (2024). Langmuir.

[cit31] Stani C., Vaccari L., Mitri E., Birarda G. (2020). Spectrochim. Acta, Part A.

[cit32] Bridelli M., Capelletti R., Mora C. (2013). J. Phys. D:Appl. Phys..

[cit33] Buontempo U., Careri G., Fasella P. (1972). Biopolymers.

[cit34] Gorr H. M., Zueger J. M., Barnard J. A. (2012). Langmuir.

[cit35] Sett A., Ayushman M., Dasgupta S., DasGupta S. (2018). J. Phys. Chem. B.

[cit36] Choi Y., Han J., Kim C. (2011). Korean J. Chem. Eng..

[cit37] Yunker P. J. , Coffee-rings and Glasses: Colloids Out of Equilibrium, University of Pennsylvania, 2012

[cit38] Deegan R. D., Bakajin O., Dupont T. F., Huber G., Nagel S. R., Witten T. A. (1997). Nature.

[cit39] Carreón Y. J., González-Gutiérrez J., Pérez-Camacho M., Mercado-Uribe H. (2018). Colloids Surf., B.

[cit40] Pal A., Gope A., Iannacchione G. S. (2019). MRS Adv..

[cit41] Pal A., Gope A., Kafle R., Iannacchione G. S. (2019). MRS Commun..

[cit42] Sempels W., De Dier R., Mizuno H., Hofkens J., Vermant J. (2013). Nat. Commun..

[cit43] Still T., Yunker P. J., Yodh A. G. (2012). Langmuir.

[cit44] Kajiya T., Kobayashi W., Okuzono T., Doi M. (2009). J. Phys. Chem. B.

[cit45] Hu H., Larson R. G. (2006). J. Phys. Chem. B.

[cit46] Deegan R. D., Bakajin O., Dupont T. F., Huber G., Nagel S. R., Witten T. A. (2000). Phys. Rev. E.

[cit47] Chen G., Mohamed G. J. (2010). Eur. Phys. J. E:Soft Matter Biol. Phys..

[cit48] Yakhno T. A. (2011). Phys. Chem. Chem. Phys..

[cit49] Annarelli C., Fornazero J., Bert J., Colombani J. (2001). Eur. Phys. J. E:Soft Matter Biol. Phys..

[cit50] Gorr H., Zueger J., McAdams D., JA B. (2013). Colloids Surf., B.

[cit51] Pathak B., Christy J., Sefiane K., Gozuacik D. (2020). Langmuir.

[cit52] Basu N., Mukherjee R. (2020). J. Phys. Chem. B.

[cit53] Pal A., Gope A., Athair A. S., Iannacchione G. S. (2020). RSC Adv..

[cit54] Pal A., Gope A., Iannacchione G. (2021). Biomolecules.

[cit55] Carreón Y. J., Ríos-Ramírez M., Vázquez-Vergara P., Salinas-Almaguer S., Cipriano-Urbano I., Briones-Aranda A., Díaz-Hernández O., Santos G. J. E., González-Gutiérrez J. (2021). Colloids Surf., B.

